# Supplementary material for: Cosmopolitan inversions have a major impact on trait variation and the power of different GWAS approaches to identify associations
Source: bioRxiv. 2025 Sep 20:2025.09.17.676858. Preprint. [Version 1] doi: 10.1101/2025.09.17.676858 (PMC12458207; doi:10.1101/2025.09.17.676858)
Supplement: Supplement 1 — Supplemental Figure 1: The addition of ancestry does not remove the broad impact of inversion genotype on phenotype. A The number of phenotypes with significant associations is shown as diamonds for the Ancestry and Inversion model, as well as for a Full model that uses both ancestry and inversion genotype as fixed effect. A set of paired 100 permutations of each model is shown as a box and whisker plot. Results are split across five cosmopolitan inversions, and colored by trait classification. B The same plot as in A, now showing a comparison between the Full and Ancestry models, as well as the Full and Inversion models. Supplemental Figure 2 Signal of loci association with In(2L)t is mostly adjacent to the inversion. The same results of the association study using the LOCO method from Fig 6 are shown across the genome, showing the likelihood of a SNP’s association with PC1, PC2, or both from the In(2L)t PCA analysis Supplemental Figure 3. Signal of loci association with In(3R)Mo is elevated on 3R. The same results of the association study using the LOCO method from Figure 6 are shown across the genome, showing the likelihood of a SNP’s association with PC1, PC2, or both from the In(3R)Mo PCA analysis Supplemental Figure 4. Factored-out method fails to identify areas of likely association. A) Results of a sliding window analysis examining enrichment between SNPs scored using Factored-out for PC1 and PC2 of In(2L)t, the yaxis shows the strength of enrichment and the x-axis shows position on the genome. Grey shaded region show the zone of cosmopolitan inversions on the chromosome arm. B) Same analysis as in A, but considering In(3R)Mo. [file media-1.docx]

## Supplemental Figures


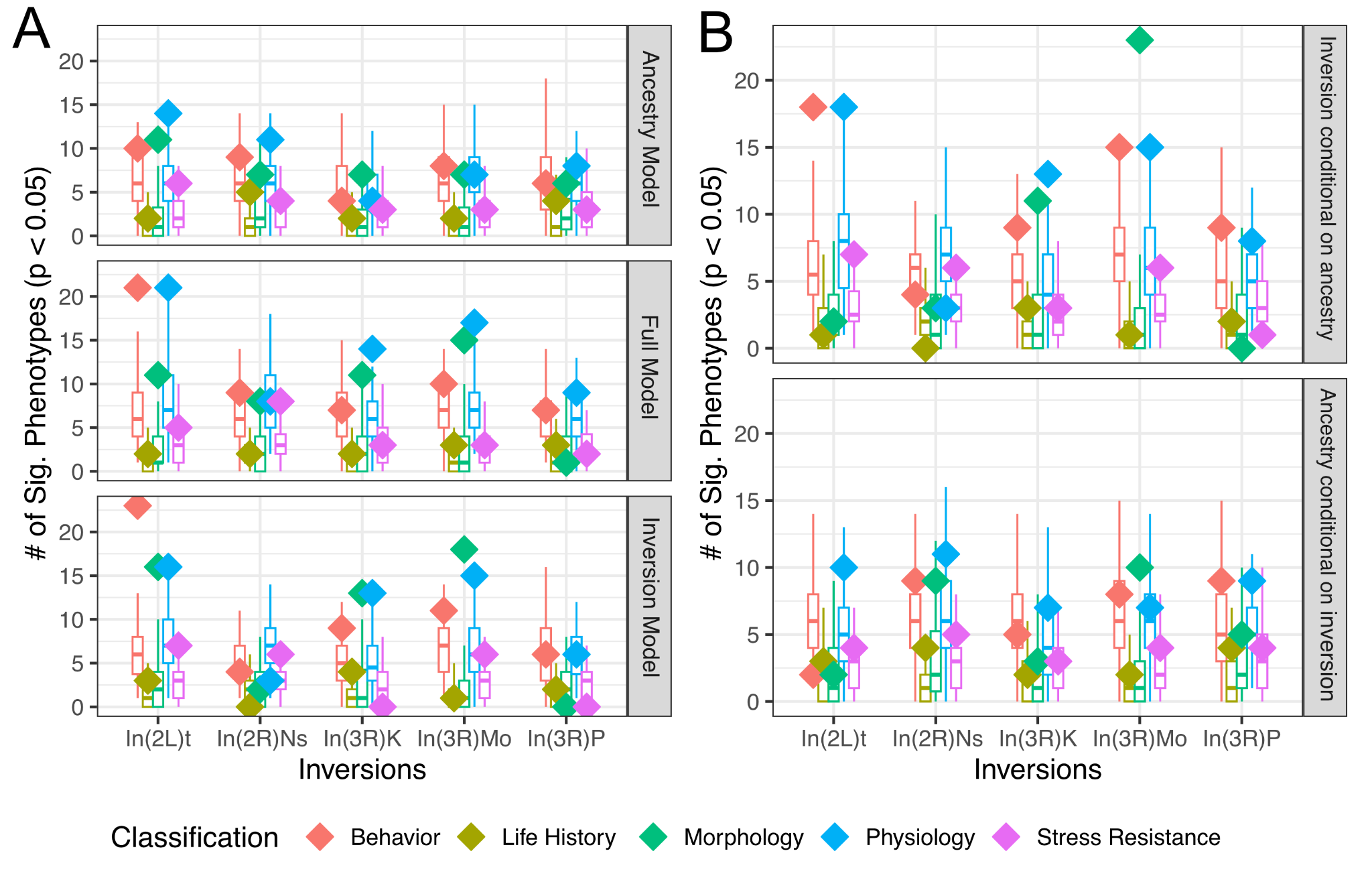


**Supplemental Figure 1: The addition of ancestry does not remove the broad impact of inversion genotype on phenotype.** A The number of phenotypes with significant associations is shown as diamonds for the Ancestry and Inversion model, as well as for a Full model that uses both ancestry and inversion genotype as fixed effect. A set of paired 100 permutations of each model is shown as a box and whisker plot. Results are split across five cosmopolitan inversions, and colored by trait classification. B The same plot as in A, now showing a comparison between the Full and Ancestry models, as well as the Full and Inversion models.


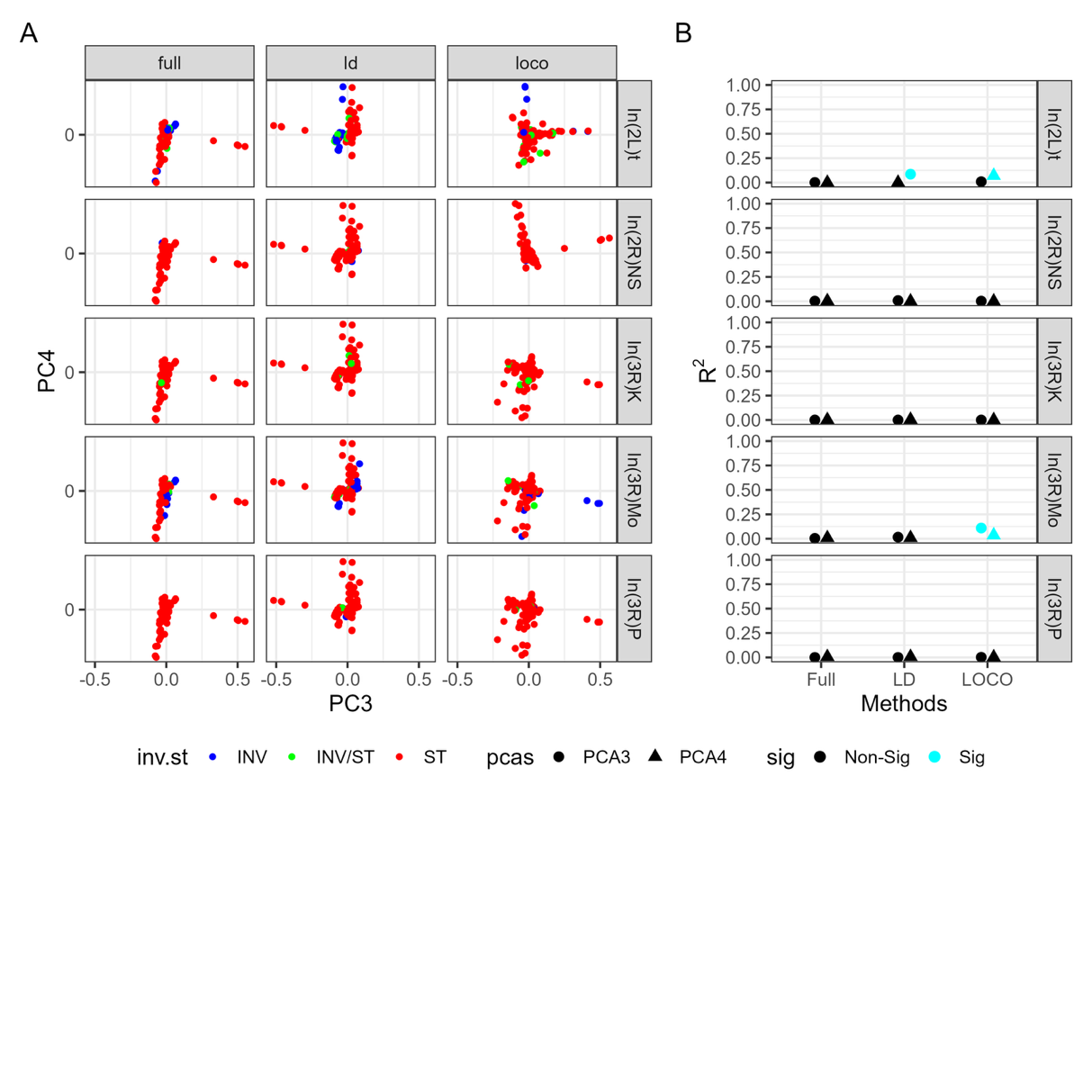


**Supplemental Figure 2:** Genomic principal components PC3 and PC4 have little correlation with inversion genotype. **A)** The third and fourth genomic PCs for each sample colored by the genotype of that sample. **B)** The R^2^ values for models comparing PC3 and PC4 to inversion, colored by which values exceed a distribution of permutations.


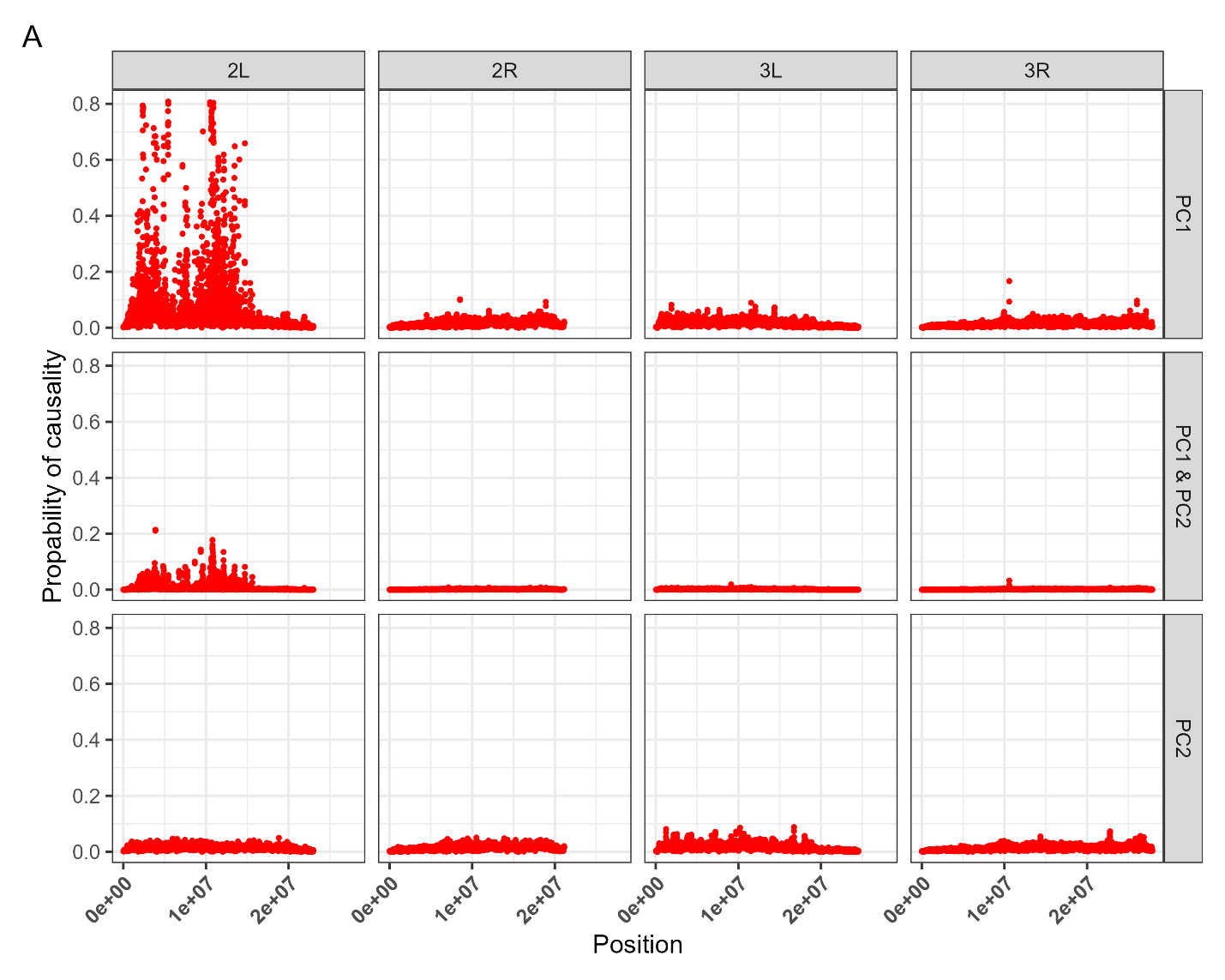


**Supplemental Figure 3.** Signal of loci association with In(2L)t is mostly adjacent to the inversion. The same results of the association study using the LOCO method from Fig 6 are shown across the genome, showing the likelihood of a SNP’s association with PC1, PC2, or both from the In(2L)t PCA analysis


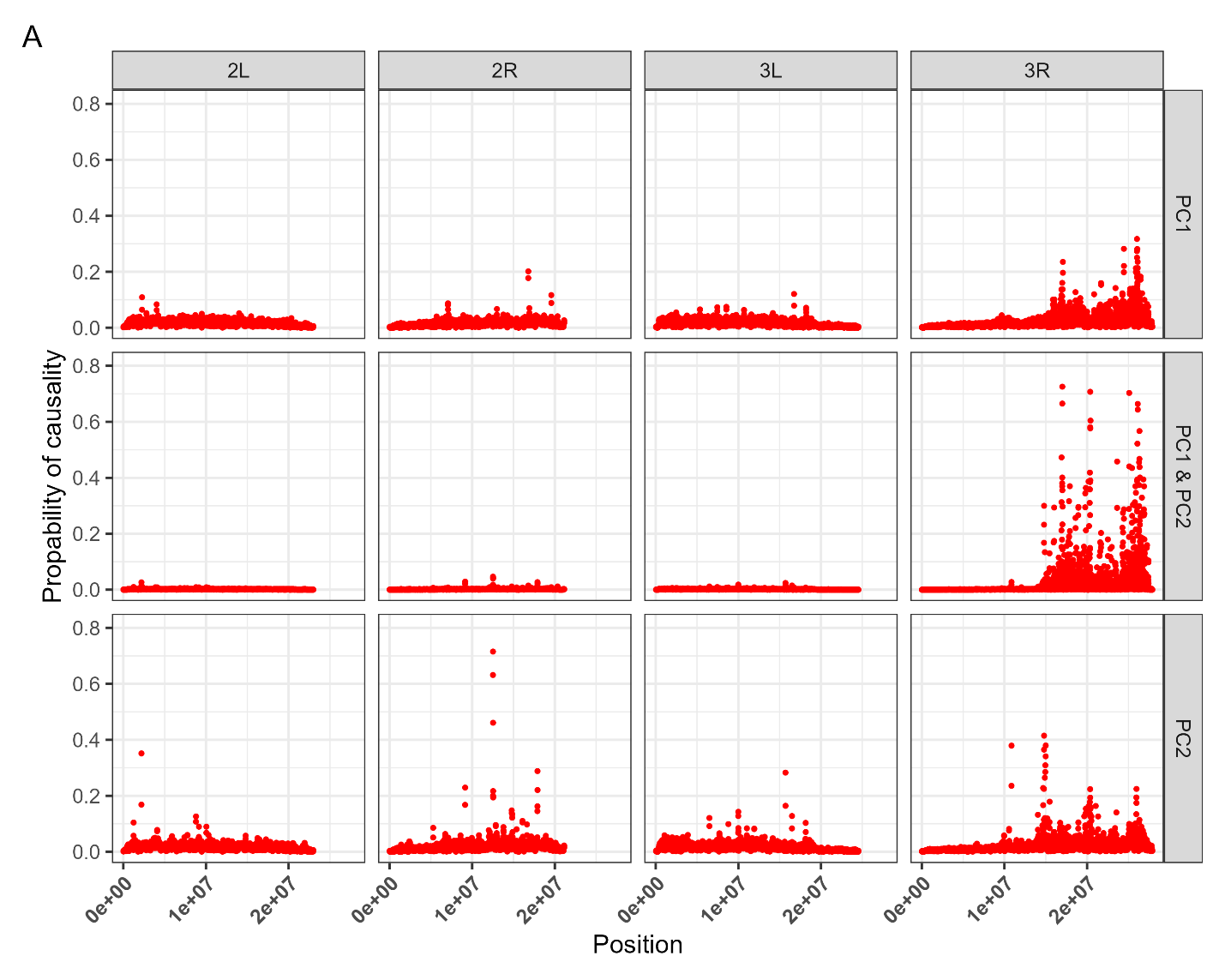


**Supplemental Figure 4.** Signal of loci association with In(3R)Mo is elevated on 3R. The same results of the association study using the LOCO method from Figure 6 are shown across the genome, showing the likelihood of a SNP’s association with PC1, PC2, or both from the In(3R)Mo PCA analysis


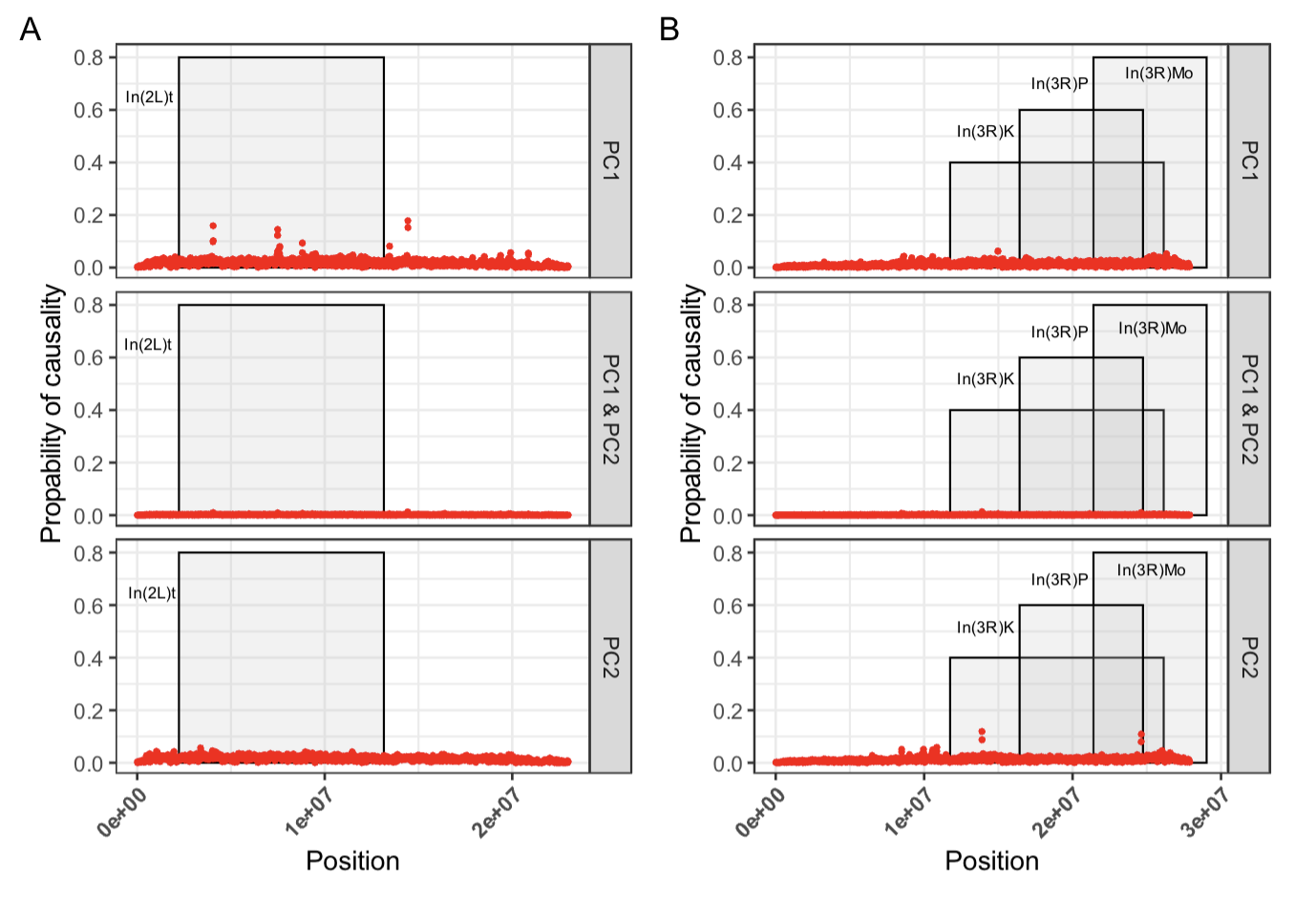


**Supplemental Figure 5.** Factored-out method fails to identify areas of likely association. **A)** Results of a sliding window analysis examining enrichment between SNPs on 2L scored using Factored-out for PC1 and PC2 of In(2L)t, the y- axis shows the strength of enrichment and the x-axis shows position on the genome. Grey shaded region show the zone of cosmopolitan inversions on the chromosome arm. **B)** Same analysis as in A, but considering chromosome arm 3R and inversion In(3R)Mo.
